# Metal-Organic Frameworks for Sensing Applications in the Gas Phase

**DOI:** 10.3390/s90301574

**Published:** 2009-03-06

**Authors:** Sabine Achmann, Gunter Hagen, Jaroslaw Kita, Itamar M. Malkowsky, Christoph Kiener, Ralf Moos

**Affiliations:** 1 Functional Materials, University of Bayreuth, Universitätsstr. 30, 95440 Bayreuth, Germany; 2 BASF SE, Ludwigshafen, Germany

**Keywords:** Metal-organic framework, MOF, impedance spectroscopy, humidity, gas sensor

## Abstract

Several metal-organic framework (MOF) materials were under investigated to test their applicability as sensor materials for impedimetric gas sensors. The materials were tested in a temperature range of 120 °C - 240 °C with varying concentrations of O_2_, CO_2_, C_3_H_8_, NO, H_2_, ethanol and methanol in the gas atmosphere and under different test gas humidity conditions. Different sensor configurations were studied in a frequency range of 1 Hz -1 MHz and time-continuous measurements were performed at 1 Hz. The materials did not show any impedance response to O_2_, CO_2_, C_3_H_8_, NO, or H_2_ in the gas atmospheres, although for some materials a significant impedance decrease was induced by a change of the ethanol or methanol concentration in the gas phase. Moreover, pronounced promising and reversible changes in the electric properties of a special MOF material were monitored under varying humidity, with a linear response curve at 120 °C. Further investigations were carried out with differently doped MOF materials of this class, to evaluate the influence of special dopants on the sensor effect.

## 1. Introduction

Metal-organic framework materials (MOF) are well-known since they can store large amounts of hydrogen [1,2] or can be applied for gas purification [3]. These applications are based on their high specific surface area, which is a result of their high and ordered porosity. As small molecules like hydrogen are only adsorbed and not covalently bound to the surface, they can be released completely, for example at lower partial pressures. Furthermore, catalysis is another potential field of application for MOFs [3,4]. Due to the highly modular synthesis of MOF structures, their chemical as well as morphological properties can be modified with respect to the desired application. Thus, MOFs with functionalized cavities and tailored pore sizes are accessible. Moreover, catalytic properties can be implemented by active metal centers, which simultaneously serve as nodes for the coordination framework and as reaction centers for the catalyzed reaction itself. Reference [4] reviews several catalysis applications including Ziegler-Natta polymerization, Diels-Alder reaction, photoreactions, etc.

MOFs were shown to possess superior sorption properties compared to classical sorption materials. For example, the storage capacities of zeolites and activated carbon were exceeded by almost a factor of two for carbon dioxide [5] and hydrogen [3], respectively, by use of specific MOFs. MOFs offer several advantages over zeolites and activated carbon such as their modularity, higher porosity and functionality. Still, most metal-organic frameworks are thermally and chemically significantly less stable than zeolites, so applications at high temperatures (> 400 °C) and under chemically extremely severe conditions are still reserved to purely inorganic sorption materials.

The reversible sorption behavior suggests the investigation of MOF materials for gas sensor purposes. The change of the dielectric properties of the materials, caused by adsorption or desorption of molecules on the inner surface of the MOF, might be utilized to detect selectively small amounts of gaseous analytes by measuring the electrical impedance of the material. For the first time, this study investigates MOF materials for gas sensing.

In particular, the ability to detect different amounts of humidity in gas atmospheres with MOF materials was studied. It is known from several examples reported in the literature that hydration-dehydration of MOFs can significantly change the 3D-structure of the metal-organic framework. For MIL-53, an aluminum terephthalate, a breathing effect was observed upon hydration-dehydration with a compressed structure for the dehydrated MOF [6]. A similar observation was made for the nanoporous coordination polymer [Ag_6_Cl(atz)_4_] (atz = 3-amino-1,2,4-triazolate) [7]. Here, hydration-dehydration induces a remarkable crystal interconversion between two different tetragonal structures. Adsorption of ethanol from the vapor phase was investigated using a Ni-based MOF [8]. Interestingly, only slight structural readjustment upon sorption and desorption of the simple alcohol was found.

Humidity is of great importance in a wide field of domestic and industrial applications, e.g. the quality control of production processes or an intelligent control of the living conditions in buildings [9]. From this multiple applications, different requirements arise for humidity sensors, e.g., operating temperature, humidity range, solvent stability [10]. To meet these requirements, a wide variety of sensor mechanisms was investigated for the detection, e.g., optical, resistive, capacitive, piezoresistive, gravimetric, or magnetoelastic [11]. The materials used for these sensors mainly originate from the ceramic, polymer, and semiconducting materials class [12], whereas metal-organic framework materials have not yet been investigated for this purpose to our knowledge.

The sensing mechanism of the different materials is mainly based on physical adsorption or absorption of water molecules from the surrounding atmosphere causing a change in the electrical properties (impedance or resistance and capacitance) of the sensing material [13].

Each of these sensor types suffers from limitations, so intensive research in this field continues. The main drawback of these sensors is that they are mostly only designed to detect relative humidity (rH) in the range of 10 – 90 % rH and to work at room temperature or slightly above [14]. Ceramic humidity sensors have to deal with the formation of stable chemisorbed OH^−^ at their surface. To avoid a drift in the resistance of those ceramic sensors induced by chemisorption, a periodic heat treatment at temperatures higher than 400 °C is necessary to regenerate the sensor surface [15]. Hence, ceramic humidity sensors are often equipped with a supplementary heater for this regeneration process causing extra cost and complexity [16]. This heat cleaning is not necessary for polymer sensors and their fabrication cost is much lower than for ceramic ones, as they do not need high-temperature processing [17]. However, polymers often lack thermal or chemical stability and cannot be used as humidity sensors in harsh environments. Moreover, polymer film sensors show slow response times, hysteresis and long-term drift when exposed to some solvents [10]. Even the use of metal-oxides, although thermally resistant, is restricted to oxygen rich, non reducing atmosphere. For high temperature applications above 400 °C in reducing conditions, zeolites have been investigated recently [14], but there is still a lack of sensors for medium temperature application from 100 – 300 °C [10].

The tested MOF materials are stable in a temperature range up to 250 °C [18]. As the production and processing of MOF materials is rather cheap and adsorption and desorption phenomena of water can be assumed from their surface characteristics and chemical properties as depicted above, these materials are investigated as new materials class for humidity sensors.

## 2. Experimental

### 2.1. Sensor materials

Metal-organic frameworks (MOFs) are coordination polymers composed of metal ions and organic linkers. Within the framework, the organic linkers act as bridging ligands between the metal ions. Depending on the vacant sites on the metal ion and the connectivity of the organic linker, 1-, 2- and 3-dimensional polymers can be formed. References [4] and [19] review the general aspects of porous coordination polymers and MOFs.

Three basic MOF materials were tested including Al-terephthalate-MOF (Al-BDC, Basolite™ A100), Fe-1,3,5-benzenetricarboxylate-MOF (Fe-BTC), and Cu-1,3,5-benzenetricarboxylate-MOF (Cu-BTC). In Addition, Li-doped (Fe-BTC*ld*) and Fe(II)-doped (Fe-BTC*fd*) Fe-1,3,5-benzene-tricarboxylate-MOFs were investigated in the sensing experiments. According to thermogravimetric analyses, the tested MOF materials are stable in a temperature range up to 250 °C [18]. The described MOFs were chosen due to practical reasons: all of them can be synthesized on an industrial scale starting from readily available chemicals. Thus, the MOFs presented herein are available for reasonable prices and can be produced even on large scales. Al-BDC, Cu-BTC, and Fe-BTC can be purchased as BASF-products Basolite™ A100, Basolite™ C300, and Basolite™ F300 from Sigma-Aldrich.

#### Fe-BTC*ld*

A 100 mL electrochemical beaker cell equipped with an iron anode and a steel cathode (each with an active electrode surface of 2 cm × 5 cm; electrode gap of 1 cm) was charged with a solution of 1,3,5-tricarboxylic acid (960 mg, 4.3 mmol) and methyltributylammonium methylsulfate (960 mg, 60 weight-% in methanol) in methanol (62.25 g). After warming the electrolyte to 32 °C electrolysis was performed at 3.8 A/dm^2^. After 55 minutes, a charge of 3 F/mol 1,3,5-tricarboxylic was passed and the electrolysis was stopped. Weight loss at the anode indicated anodic dissolution of 66 mmol Fe(II). The resulting brown suspension was kept at 32 °C and LiPF_6_ (100 mg) was added. After stirring for 12 h at ambient air the suspension brightened up and was filtered, washed with methanol (15 mL), and dried in vacuum at 120 °C for 12 h. Yield: 1.3 g; Langmuir surface area: 1454 m^2^/g; elemental analysis: 22% Fe, 32.6% C, 42.3% O, 0.32% S, <0.5% N, 3.1% H, 0.03% Li.

#### Fe-BTC*fd*

Fe-BTC (3 g; Langmuir surface area 1295 m^2^/g) and FeSO_4_ × 7 H_2_O (150 mg) were finely ground in a mortar for 10 minutes under an Ar-atmosphere.

### 2.2. Sensor preparation

**Figure 1.**
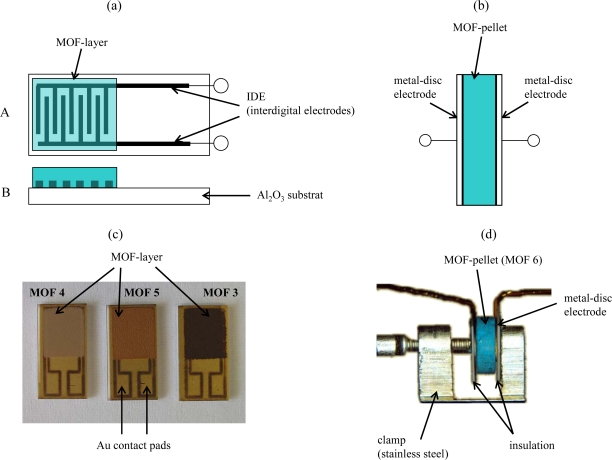
Sensor set-up. Left side: Planar sensor in thick-film technology, MOF paste screen-printed on laser-patterned interdigital electrodes (IDE), (a) schematic top view and cross section, (c) top view of real sensors with MOF materials (Fe-BTC*ld*, Fe-BTC*fd*). Right side: Pellet-type MOF-sensor with metal-disc electrodes, (b) schematic view, (d) photography of a sensor, the two metal-disc electrodes were pressed to the sides of the pellet by an adjustable screw in a clamp.

The five types of metal organic frameworks (Al-BDC, Fe-BTC, Cu-BTC, Fe-BTC*ld*, and Fe-BTC*fd*) were provided in powder form or in pellet form by BASF. From these materials, two different sensor set-ups were prepared as described in Figure 1. The powders were ground, mixed with an organic vehicle (KD2721, Zschimmer & Schwarz) and processed with a three-roll mill to achieve printable thick-film pastes. For the planar sensor set-up, interdigital electrodes (IDE) were prepared as transducers by laser patterning of fired Au thick-film layers. For that purpose, a Au thick-film paste (QG150, DuPont) was printed on top of an alumina substrate (96%, Ceramtec 708S) and fired for 10 min at 850 °C (heating and cooling rate 30 °C/min). The obtained film was dense, had a fired thickness of approx. 10 μm and covered all over the substrate. The applied frequency-tripled Nd:YAG laser (LPKF, Germany) had a beam diameter of about 20 μm. Due to laser energy fluctuations and the surface roughness of the substrate, interdigital electrodes structures with minimal possible line widths and spaces of about 25 to 30 μm each are possible. In order to avoid short circuiting of the IDE fingers and to guarantee an almost infinite resistance between the IDE fingers, structures with 50 μm lines and 50 μm spaces were used as transducers. Further details of the laser processing can be found in [21].

To study the applicability of the different MOF materials as sensor materials, the prepared MOF-thick-film pastes were screen-printed on top of the laser patterned IDEs. Subsequently, the devices were heated to 200 °C for 40 min with a heating and cooling rate of 1 °C/min to evaporate the organic vehicle constituents. Contact to the measurement equipment was provided by two Au-wires, welded on the Au contact pads of the IDE structures (Figures 1a and 1c).

The pellets (Ø *=* 6 mm, *d =* 2 mm) were used as obtained and contacted by metal-discs (Ø *=* 6 mm) giving the pellet-shaped sensor set-up. The metal discs were equipped with a flat electrode at each single end, which were connected by Au-wires to provide a conducting interface to the measurement equipment (Figures 1b and 1d).

### 2.3. Sensor characterization

Both sensor configurations were tested between 120 °C and 240 °C in a tube furnace set-up similar to the one described in [22]. Different gas components (10 % O_2_, 10 % CO_2_, 1,000 ppm C_3_H_8_, 1,000 ppm NO, 1,000 ppm H_2_, 0 – 18 % ethanol, 0 – 35 % methanol) were admixed to the carrier gas N_2_ to evaluate the impedimetric response of the sensors. Humidity was varied in the range of 0 – 3 %vol. H_2_O of the test gas by either saturating the carrier gas with water in fritted wash-bottles in a temperature controlled water bath similar to [23] or directly by admixing different amounts of water vapor to the test gas.

The impedance spectra were recorded by a Novocontrol Alpha-Analyzer in the frequency range from 1 Hz to 1 MHz as described in [24]. Impedance spectroscopy is a useful tool to characterize the electric properties of highly resistive materials. The complex impedance *Z* consists of |*Z*|, the absolute value of *Z* and the phase angle between current and voltage φ, with Re(*Z*) = *Z′* and Im(*Z*) = *Z*″. If one assumes that the system consists of discrete elements, one may use a parallel equivalent circuit of resistor *R* and capacitor *C*. Then, the two independent measurands Z′ and Z″ can be expressed by *R* and *C*. Further details can be found in the standard reference book [25].

In addition to the measurements in the frequency range stated above, the complex impedance at 1 Hz was continuously measured to determine the sensor response to stepwise changed gas compositions. The gas composition was monitored by an FTIR analysis system (Nicolet 6700, Thermo) downstream of the tube furnace. An example for a special test procedure at 1 Hz to evaluate the sensor characteristics for varying humidity is given in Table 1.

Two devices were prepared of each material and sensor set-up to evaluate the reproducibility of the sensor response. The repeatability of the signal was determined when the sensors were subjected to the same test procedure for several times.

## 3. Results and Discussion

Not depending on the admixed concentrations of non-polar gases to the carrier like O_2_, C_3_H_8_, and H_2_, the impedance of all MOF materials remained unaffected. NO and CO_2_ also did not lead to changing impedances.

**Figure 2.**
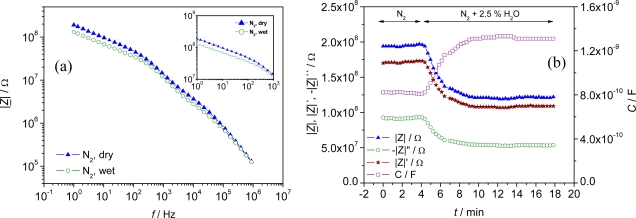
Impedimetric sensor response to variations in the humidity of the test gas. Pellet-type sensor of Al-BDC. (a) Measurements in a frequency range between 1 Hz - 1 MHz in dry and wet N_2_ carrier gas. (b) Time dependent measurements at 1 Hz when the surrounding gas is varied stepwise from dry N_2_ to N_2_ with 2.5 vol% H_2_O. *T* = 120 °C.

Two of the selected metal organic frameworks (Al-BDC and Fe-BTC) responded to different humidity concentrations of the carrier gas. For example, a decrease in the impedance was detected for a pellet sensor of Al-BDC when the gas flow was changed from dry to wet (2.5 vol.% H_2_O) at 120 °C (Figure 2a). As obvious from this Figure, the difference in the impedance between dry and wet carrier gas increases towards lower frequencies. Therefore, a frequency of 1 Hz was used to evaluate the influence of varying humidity on the impedance of the sensor in time-dependent measurements (Fig. 2b).

As can be only poorly resolved from Figure 2a, the sensor showed a strong response in all impedance-derived measurands (|*Z*|, *Z*′, *Z*″, and *C*) at 1 Hz. For instance, the absolute value |*Z*| changed by 39 % of the base impedance in N_2_ (∼ 76 MΩ), when the surrounding atmosphere was changed from dry N_2_ carrier gas to humidified gas (2.5 vol% H_2_O), meaning a sensitivity of 30.4 MΩ/vol%H_2_O. The sensor showed stable signals both in dry and wet carrier gases within one measurement cycle.

**Figure 3.**
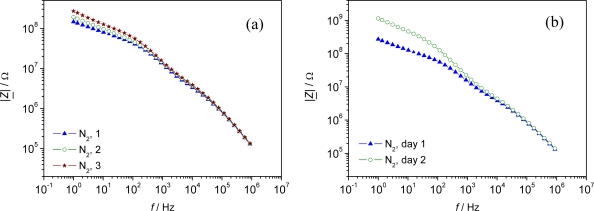
Reproducibility of the impedimetric sensor signal (|*Z*|) in N_2_ gas atmosphere. Frequency range 1 Hz - 1 MHz. Pellet-type sensor of Al-BDC. (a) Repeatability of the sensor characteristics in three consecutive measurements. (b) Repeatability at two consecutive days. All measurements performed with the same pellet. *T* = 120°C.

However, a strong shift in the base impedance in N_2_ was observed, when those pellets were tested consecutively within one day (Figure 3a). An even stronger shift was observed when the impedimetric response was evaluated at two consecutive days (Figure 3b). This shift is supposed to be due to a change in the MOF-characteristics itself, but has to be evaluated in more detail in further studies.

**Figure 4.**
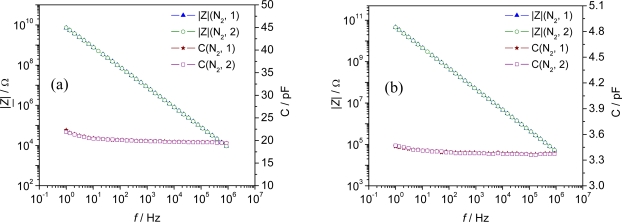
Reproducibility of the impedimetric sensor signal (|*Z*| and *C*) in N_2_ gas atmosphere in two consecutive measurements. Frequency range 1 Hz - 1 MHz. (a) Screen-printed planar IDE sensor configuration with Fe-BTC. (b) Pellet-type sensor of Fe-BTC. *T* = 120 °C.

As Al-BDC did not show reproducible results in the base signals, and even the impedimetric response to humidified test gas varied from test cycle to test cycle, it was not investigated any further. Unlike Al-BDC sensors, sensor elements made of Fe-BTC showed significantly higher and repeatable effects when they were exposed to a varying humidity. Advantageously, neither the screen-printed sensor configuration (Figure 4a) nor the pellet-type set-up (Figure 4b) showed any significant changes in the base impedance or capacitance in N_2_ carrier gas in consecutive measurements (Fig. 4).

**Figure 5.**
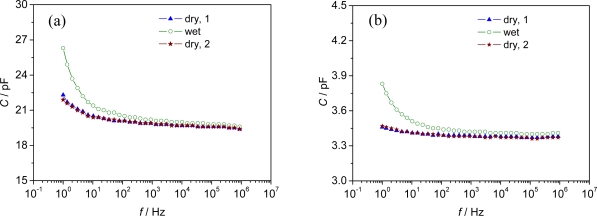
Sensor signal (changing capacitance *C*) of Fe-BTC sensors at 120 °C in dry and H_2_O saturated N_2_ gas atmosphere (saturated at 20 °C, ∼ 2.3 %vol.). (a) IDE sensor set-up, (b) pellets sensor. Measurement range 1 Hz - 1 MHz at 120 °C.

Best results, with a reversible change in the absolute value of the complex impedance |*Z*| and capacitance *C*, were obtained for the planar IDE sensors. When the humidity of the carrier gas at 120 °C was changed from dry to wet (∼ 2.3 vol%, H_2_O saturated at 20 °C) the capacitance of the planar IDE sensor increased by 4.4 pF (20.1 % of the base capacitance in N_2_) and the capacitance of the pellet-type sensor by 0.36 pF (10.4 % of the base capacitance), respectively, at low frequencies (Figure 5).

Again, an increasing difference between dry and wet (2.3 %vol.) carrier gas was observed with decreasing measurement frequency for C and |*Z*|. Hence, a frequency of 1 Hz was chosen for the time-dependent measurements at different temperatures between 120 °C and 240 °C.

A decreasing base impedance of the planar Fe-BTC sensors was observed in N_2_ carrier gas with an increase in the sensor temperature. By comparing two different but equally manufactured sensors one recognizes the good reproducibility, which is considerably high at 120 °C (Table 2).

**Figure 6.**
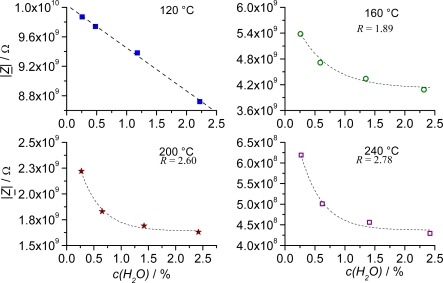
Response curves (|*Z*| vs. c(H_2_O)) of a planar Fe-BTC IDE sensor at different humidity of the test gas (0 – 2.5 vol%). The measurements were conducted at four different temperatures: 120 °C, 160 °C, 200 °C, and 240 °C. The sensor characteristics changed from a linear dependence at 120 °C to a behavior that can be approximated by exponential decay. Measurement frequency: 1 Hz.

It was also noted that the sensitivity to variations in the water content of the test gas decreased with increasing temperature. At 120 °C, an almost linear behavior was observed (Figure 6).

For increasing temperature, an exponential decay in the form:

(1) $$\left|\underline{Z}\right|=\left|\underline{Z_{0}}\right|+A\cdot e^{\left(-R\cdot c\left(H_{2}O\right)\right)}$$

can be used to approximate the dependence of the sensor response |*Z*| from the water content *c*(H_2_O) of the test gas with the exponential rate *R* and an initial value *A*. The exponential rate *R* increases in the fit curves with increasing temperature up to 200 °C. That can be attributed to a faster rate of saturation of the MOF-material or an increasing influence of the desorption process. This increase of desorption with temperature will also be demonstrated in the sensor characteristics by a faster saturation of the sensor signal at high water content (Figure 6).

**Figure 7.**
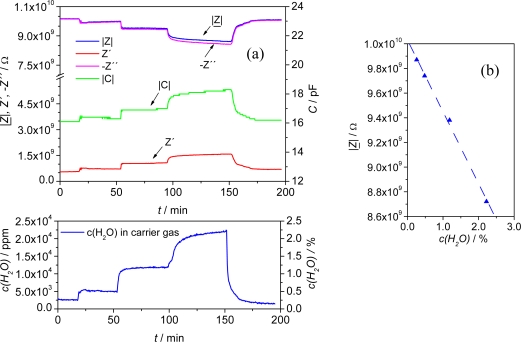
(a) Upper part: Impedimetric sensor signal (|*Z*|, Z′, -Z″, C) of an IDE equipped with Fe-BTC in time-continuous measurements at 1 Hz for 0 – 2.5 vol%H_2_O in the test gas. Lower part: H_2_O concentration in the test gas over time evaluated by FTIR. (b) Response curve of the sensor (|*Z*| vs. c(H_2_O)). At 120 °C a linear dependence of the complex impedance from the humidity of the test gas was observed with a sensitivity of 590 MΩ/vol%H_2_O.

At a temperature of 120 °C, an overall impedance increase of 1.2 GΩ and a linear sensitivity of 590 MΩ/vol% H_2_O was achieved in a humidity range of 0 to 2.5 vol% (Figure 7).

If one defines a relative sensitivity by the relative change of the absolute value of the impedance at the fixed frequency of 1 Hz when exposed to 2.5 vol% H_2_O normalized to the initial value in dry gas:

(2) $$S_{H_{2}O}=\frac{\left|\underline{Z}\left(c_{H_{2}O}\right)\right|-\left|\underline{Z}\left(c_{H_{2}O}=0\right)\right|}{\left|\underline{Z}\left(c_{H_{2}O}=0\right)\right|}\cdot \frac{1}{c_{H_{2}O}}\cdot 100\%$$

one obtains *S*_H_2_O_ = 5.4%/vol%H_2_O.

After having the sensor exposed to different H_2_O-concentrations, the impedance signal recovered almost completely to the initial value, with a marginal shift of 0.4 % of the initial impedance in N_2_. With this extremely low shift MOF materials perform very well compared to e.g., thin film polymer sensors [13].

Due to this high reproducibility and sufficient sensitivity to variations in humidity, this MOF material showed quite promising properties for gas sensor applications. Thus, the effect of other hydrophilic gases like ethanol and methanol in the gas phase on the impedimetric properties of planar Fe-BTC-sensors was evaluated.

**Figure 8.**
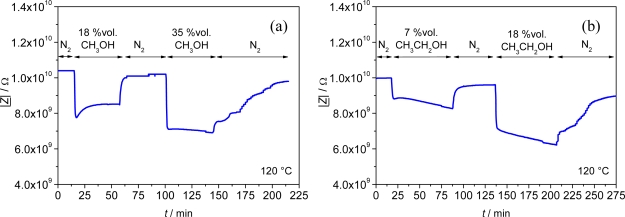
(a) Impedimetric sensor signal (|*Z*|) of a planar Fe-BTC sensor at 1 Hz for 0 – 35 vol% methanol in the test gas. (b) Impedimetric sensor signal (|*Z*|) of the same sensor at 1 Hz for 0 – 18 vol% ethanol in the test gas. Measurement temperature: *T* = 120 °C.

Similarly to the response towards humidity changes, the complex impedance |*Z*| and capacitance *C* of planar Fe-BTC was affected by varying ethanol and methanol concentrations in the test gas, with the highest and most linear sensitivity achieved at 120 °C. However, the sensors required much more time to recover to the base impedance in N_2_ after they had been exposed to ethanol or methanol containing N_2_. A similar observation was reported in the case of methanol and ethanol sorption in a Cu(II)-framework [26]. Both, methanol and ethanol were found to show hysteric sorption isotherms, indicating a strong adsorbate-host interaction and structural readjustment of the framework upon sorption-desorption. For Fe-BTC additionally, a drifting impedance value was observed when the planar sensor was exposed to ethanol (Figure 8b).

Altogether, at 120 °C the influence of the test gases increased in the order from methanol to ethanol to H_2_O, with a relative sensitivity *S*_methanol_ = 0.99 %/vol% CH_3_OH obtained for methanol and *S*_ethanol_ = 2.3 %/vol% C_2_H_5_OH for ethanol. Most stable and best reversible signals were obtained for variations in the water content of the test gas. Cross sensitivity of the MOF-sensor on ethanol and methanol can be evaluated when a cross-sensitivity factor (*CSF*) is introduced similar to [27] comparing the relative sensor response for 1 vol% of the interfering gas species with the sensor response for 1 vol% H_2_O in the test gas.

(3) $${\mathrm{CSF}}_{\mathrm{interfering gas}}=\frac{\left|\underline{Z}\left(c_{\mathrm{interfering gas}}\right)\right|-\left|\underline{Z}\left(c_{\mathrm{interfering gas}}=0\right)\right|\cdot \frac{1}{c_{\mathrm{interfering gas}}}}{\left|\underline{Z}\left(c_{H_{2}O}\right)\right|-\left|\underline{Z}\left(c_{H_{2}O}=0\right)\right|\cdot \frac{1}{c_{H_{2}O}}}$$

If a *CSF* > 1 is derived from eq. (3), the sensor responds more to the interfering gas than to humidity, whereas for CSF < 1 a low reactivity of the MOF-material on this gas component is determined compared to the reactivity on humidity. For methanol, one obtains *CSF*_methanol_ = 0.32 and for ethanol *CSF*_ethanol_ = 0.69, respectively. As both values are < 1 the cross sensitivity of the sensor to methanol and ethanol is rather low.

In order to optimize the sensitivity to humidity and to further investigate the influence of dopants in Fe-BTC material, lithium (Fe-BTC*ld*) and iron (Fe-BTC*fd*) were tested as dopants. The measurements were conducted at 120 °C in N_2_ carrier gas and with 1.5 vol% H_2_O admixed to the carrier gas.

Similarly to sensors with undoped material, planar sensors coated with Fe-BTC*ld* and Fe-BTC*fd* showed a good reproducibility when the impedimetric signals of two different sensors in N_2_ were compared. Even no significant changes in the absolute values compared to undoped material were observed (Table 3).

All tested materials were sensitive to humidity in the test gas. The sensitivity of the materials increased like:

$$S_{\text{Fe-BTC}\mathrm{fd}}<S_{\text{Fe-BTC}\mathrm{ld}}\leq S_{\text{Fe-BTC}}.$$

**Figure 9.**
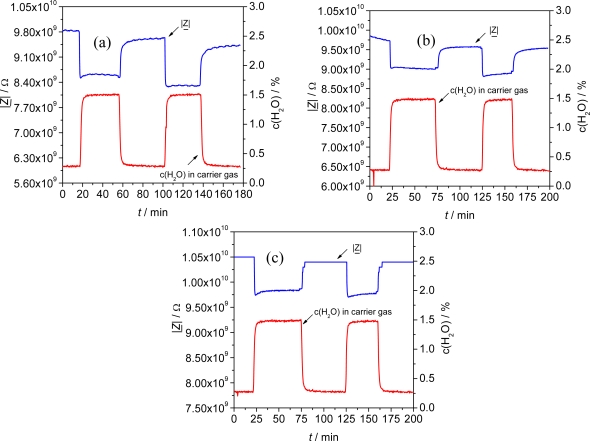
Impedimetric sensor signal (|*Z*|) of differently doped planar Fe-BTC sensors at 1 Hz. The composition of the test gas was changed from N_2_ to 1.5 vol% H_2_O consecutively. (a) Fe-BTC (undoped), *S*_1_ = 10.4 %/%volH_2_O; *S_2_* = 11.1 %/vol%H_2_O (b) Fe-BTC*ld* (Li-doped), *S*_1_ = 6.2 %/%volH_2_O; *S_2_* = 5.9 %/vol%H_2_O (c) Fe-BTC*fd* (Fe-doped), *S*_1_ = 5.1 %/%volH_2_O; *S_2_* = 5.4 %/vol%H_2_O. Sensor temperature: 120 °C.

Thereby, the reproducibility of the impedimetric sensor signal |*Z*| to H_2_O was higher than 93 % for all materials in two consecutive measurements (Figure 9). Moreover, the impedance signal of the doped materials almost completely recovers to the initial value in N_2_ after exposure to humidity. For example, Fe-BTC*fd* shows a recovery of the material of 99.5 % and 99.7 % respectively, relating to the initial impedance in N_2_ for two different sensors (Figure 9c).

In conclusion, doping the base material did not have a significant effect on the main properties of the material as impedimetric sensor material. The materials still showed reproducible results and the same base impedance in N_2_. Only the sensitivity to H_2_O was slightly affected but remained present.

## 4. Conclusions

Different materials from the metal-organic frameworks class have been investigated for the first time as sensor materials for impedimetric humidity sensors. Two different sensor set-ups have been designed and studied in different gas atmospheres at temperatures between 120 °C and 240 °C. Most of the materials did not show any sensor effect when probed with nitrogen that contained O_2_, CO_2_, C_3_H_8_, NO, H_2_, ethanol or methanol or a different water contents. However, one material (Fe-BTC) was identified as an appropriate sensor material for the detection of hydrophilic gases like ethanol, methanol or humidity. It did not show a cross sensitivity towards O_2_, CO_2_, C_3_H_8_, NO, and H_2_. The sensitivity towards ethanol and methanol was by far lower than for water. Hence, a selective online humidity sensor was successfully manufactured by screen-printing this material on ceramic transducers with laser patterned interdigital electrodes. The absolute value of the complex impedance or the capacitance of the sensor at 1 Hz were good measurands. Best results were obtained at 120 °C with a linear response curve in the tested humidity range of 0 to 2.5 vol% and a sensitivity of 590 MΩ/vol% H_2_O (5.4 %/vol% H_2_O). The recovery behavior of the tested material after exposure to humidity was highly promising. Hence, additional heater structures as required for ceramic devices to regenerate the sensor surfaces seem to be unnecessary. It is expected that the device can be further developed to a long-term stable water vapor sensor. Differently doped versions of this material showed a high sensitivity to humidity as well, but did not lead to a better sensor performance. It should be noted that the sensors show a remarkable initial reproducibility with respect to sample scattering.

On the way to a serial product, besides material optimization, miniaturization efforts should be carried out, for instance by applying the sensor to ceramic or silicon micro hot-plates, e.g. as shown in Refs. [28] or [29], respectively. Then, the high ohmic behavior of the material needs special attention, since in this case, if the IDE resolution remains unchanged, one has to deal with sensor impedances in the GΩ-range, due to the reduced IDE area of the micro hot-plates. As a conclusion, one has to use photolithographically defined interdigital electrodes with a resolution in the range of some μm. The latter may even make sense for the above-shown ceramic substrates in order to reduce the sensor impedance to an order, at which measurement circuits are low-cost devices.

## Figures and Tables

**Table 1.** Exemplary test procedure to evaluate the impedimetric sensor response of MOF materials at different H_2_O concentrations in N_2_ carrier gas. Flow rate: 300 mL/min. Temperature range: 120 °C – 240 °C.

**gas composition**	***t* / min**
N_2_	15 - 20
N_2_ + 0.5 vol% H_2_O	30 - 40
N_2_ + 1.5 vol% H_2_O	30 - 45
N_2_ + 3.0 vol% H_2_O	30 - 45
N_2_	>60

**Table 2.** Complex impedance |*Z*| in N_2_ carrier gas at different measurement temperatures for two different planar Fe-BTC sensors measured at 1 Hz. Impedance of the second sensor in parentheses.

T / °C	|*Z*|_N_2__ / GΩ
120	10 (9.9)
160	5 (5.4)
200	2.5 (2.2)
240	0.9 (0.6)

**Table 3.** Impedance |*Z*| in N_2_ carrier gas for differently doped Fe-BTC materials at 120 °C. For each material composition, two equally prepared sensors were compared at 1 Hz. Impedance of the second sensor in parentheses.

MOF material	|*Z*|_N_2__ / GΩ	*S* / %/%volH_2_O
undoped (Fe-BTC)	10 (9.9)	8.6 (10.4)
Li doped (Fe-BTC*ld*)	10.1 (9.7)	9.4 (6.2)
Fe doped (Fe-BTC*fd*)	9.6 (10.1)	3.8 (5.1)
